# Evaluation of Implementation of Intermittent Screening and Treatment for Control of Malaria in Pregnancy in Jharkhand, India

**DOI:** 10.4269/ajtmh.19-0514

**Published:** 2020-03-09

**Authors:** Jayne Webster, Vinit Kumar Mishra, Anupkumar R. Anvikar, Irene Kuepfer, Jenna Hoyt, Jane Bruce, Brian Greenwood, Daniel Chandramohan, Neena Valecha, Neelima Mishra

**Affiliations:** 1 London School of Hygiene and Tropical Medicine, London, United Kingdom;; 2 National Institute for Malaria Research, Delhi, India

## Abstract

This study evaluated intermittent screening and treatment during pregnancy (ISTp) for malaria using rapid diagnostic tests (RDTs) at antenatal care (ANC) compared with passive case detection within the routine health system. The mixed-method evaluation included two cross-sectional household surveys (pre- and post-implementation of ISTp), in-depth interviews with health workers, and focus group discussions (FGDs) with pregnant women. Differences in proportions between surveys for a number of outcomes were tested; 553 and 534 current and recently pregnant women were surveyed (pre- and post-implementation, respectively). In-depth interviews were conducted with 29 health providers, and 13 FGDs were held with pregnant women. The proportion of pregnant women who received an RDT for malaria at ANC at least once during their pregnancy increased from pre- to post-implementation (19.2%; 95% CI: 14.9, 24.3 versus 42.5%; 95% CI: 36.6, 48.7; *P* < 0.0001), and the proportion of women who had more than one RDT also increased (16.5%; 95% CI: 13.1, 20.5 versus 27.7%; 95% CI: 23.0, 33.0; *P* = 0.0008). Post-implementation, however, only 8% of women who had completed their pregnancy received an RDT on three visits to ANC. Health workers were positive about ISTp mainly because of their perception that many pregnant women with malaria were asymptomatic. Health workers perceived pregnant women to have reservations about ISTp because of their dislike of frequent blood withdrawal, but pregnant women themselves were more positive. Intermittent screening and treatment during pregnancy was not sufficiently adopted by health workers to ensure the increased detection of malaria infections achievable with this strategy in this setting.

## INTRODUCTION

Despite best efforts and tremendous progress, malaria continues to infect an estimated 212 million people annually, leading to approximately half a million deaths.^1^ It is estimated that in 2015, 89% of malaria cases in the Southeast Asian region occurred in India,^1^ up from 50% in 2004.^2^ Malaria in pregnancy (MiP) has devastating consequences for both the mother and baby. An estimated 88.2 of 125.2 million pregnancies (70%) in malaria-endemic regions occur in the Asia-Pacific region each year, of which 28.2 million of those at risk of *Plasmodium falciparum* malaria and 32.9 million of those at risk of *Plasmodium vivax* occur in India.^3^ The clinical effects of MiP depend on the level of transmission, the malaria species, and the level of immunity in pregnant women. Both *P*. *falciparum* and *P*. *vivax* contribute to the burden of MiP in India. *P. falciparum* malaria infections in pregnancy are associated with severe maternal anemia, fetal loss, and low birth weight (LBW), whereas *P. vivax* is associated with maternal anemia, LBW, and preterm births.^4–6^

The intensity of malaria transmission is relatively low in most malaria-endemic areas of India, and subsequently, the level of acquired immunity is low. Malaria in pregnancy in India is reported to be usually symptomatic, but it can cause severe maternal malaria, death, and/or fetal loss in addition to maternal anemia and LBW.^7^ In a recent study in Jharkhand, the risk of MiP was found to be relatively low based on an assessment of the prevalence of placental malaria of 3.6% in rural and 0.8% in urban areas, but approximately 20% of women with a febrile illness were positive for malaria parasites.^8^

Currently, the policy for control of MiP in India is passive case detection (PCD). The use of long lasting insecticidal nets (LLINs) is recommended in high-risk areas, but no formal delivery system for LLINs is linked with antenatal care (ANC) services. Under this policy, symptomatic pregnant women are tested for malaria parasites and microscopy-positive women are treated according to the national policy. This strategy relies on pregnant women being symptomatic for malaria and on their reporting of their symptoms either through treatment seeking or on visits to an antenatal clinic. Contrary to previous beliefs, however, it is now apparent that a high proportion of malaria infections in pregnant women in low-transmission settings of Asia are asymptomatic.^9–11^ A potential alternative strategy is intermittent screening and treatment during pregnancy (ISTp). Intermittent screening and treatment during pregnancy involves pregnant women being screened for malaria using an rapid diagnostic test (RDT) on each visit to an ANC, regardless of symptoms, and treated if found to be positive. This strategy enables the immediate availability of results compared with PCD, where the location of malaria slide collection and reading are often different, leading to delays in the reporting of results.

This study was conducted alongside a cluster-randomized controlled trial undertaken to assess the effectiveness of ISTp compared with PCD on placental malaria.^12^ The aim of the study was to evaluate implementation of screening for malaria using RDTs at ANC within the routine health system in Jharkhand, India. The evaluation aimed to answer two main questions: first, what changes in ANC clinic attendance, screening, and/or testing for malaria occurred after implementation of ISTp; and second, what were the perspectives of health workers and pregnant women on screening for malaria at each visit to an ANC clinic.

## METHODS

### Study design.

A mixed-method study design was used to evaluate implementation of ISTp for the control of MiP in government health facilities. The methods included two cross-sectional household surveys conducted pre- and post-implementation of the intervention; in-depth interviews (IDIs) with health workers delivering the intervention; and focus group discussions (FGDs) with pregnant women eligible to receive the intervention.

### Study site.

The study was conducted in Murhu block, a subdistrict of Khunti district in Jharkhand state, India. Khunti district has a total population of 531,885^13^ and the highest concentration of tribal populations in the state. More than 90% of people live in rural areas. With hilly and forested terrain, the district’s main sources of income are rain-fed agriculture and trading of forestry products. Malaria transmission in India is highest among populations residing in tribal, hilly, difficult, and inaccessible areas.^14^ Malaria transmission in the study area is perennial but markedly seasonal, peaking from July to November after the maximum rainfall that normally occurs from July to September. The average female literacy rate is 51.3%, which is below the national average of 65.5%.^13^

In rural India, women access public ANC services through primary health centers (PHCs), subcenters, and anganwadi centers. Antenatal care services are available daily at the PHC level, weekly at the subcenter level, and monthly at anganwadi centers. Anganwadi centers are rural mother and child care centers, initially established in the 1970s to combat child hunger and malnutrition. Auxiliary nurse midwives (ANMs) typically deliver ANC services at all three levels and are supported by community health workers. These community health workers are referred to as accredited social health activists nationally and Sahhiyas in Jharkhand. Accredited social health activists were established in 2005 to bridge the gap between the health system and the community to improve maternal and child health.

Murhu block has one PHC, 18 subcenters, and 129 anganwadi centers. The Annual Health Survey in Khunti district, conducted in 2016, reported that 42.1% of pregnant women had at least four ANC visits and 12.5% of mothers had “full ANC” which is defined as at least four antenatal visits, at least one tetanus toxoid injection and iron and folic acid tablets or syrup taken for 100 or more days.^15^

### The intervention.

The ISTp intervention included 1) a 2-day training workshop on RDT-based ISTp, screening with RDTs, and administration of antimalarials for treatment of women with a positive result, which was quinine for the first-trimester and artesunate + sulfadoxine–pyrimethamine (AS+SP) for the second- and third-trimester pregnant women; 2) supplying information, education, and communication (IEC) materials including training manuals, wall charts, and job aids; and 3) in partnership with the district health management team (DHMT) supplying RDTs and first-line antimalarial treatment to district medical stores in the block.

The DHMT was responsible for supervision of ANC staff and management of the supply chain for antimalarial drugs and RDTs. After the training workshops, each ANM/subcenter was visited once by the study team to ensure the correct use of IEC material and work aids as well as to respond to unanswered questions. Introduction of ISTp into health facilities of Murhu block began with a sensitization workshop. Implementation of ISTp carried out from June 2013 to June 2014.

### Sample size.

The household surveys (see following paragraph) had multiple outcomes relating to ISTp and attendance at ANC. For each survey (pre- and post-implementation), a sample size of 540 currently and recently pregnant women was required to estimate a conservative 50% prevalence with a 95% confidence limit of ± 6%, with a design effect of 2. Recently pregnant women were included because of their exposure to the intervention over the period of its 12-month implementation and were defined as those who had delivered within the previous 12 months.

### Selection of participants and data collection.

#### Household surveys.

A two-stage cluster sampling approach was used to select 30 clusters (villages) within Murhu block based on sampling proportional to the size. The 30 clusters were enumerated to identify all currently and recently pregnant women. A maximum of 22 women were randomly sampled from each enumerated cluster. If less than 22 women were identified per cluster, all were selected.

Seventeen field workers were trained over a 1-week period on enumeration of households, informed consent procedures, and conducting and piloting the survey questionnaire. Trained field-workers were divided into eight teams, each consisting of one male and one female. Data were collected over a 3-week period between April and May 2013 for the pre-implementation survey and between April and May 2014 for the post-implementation survey. After written informed consent had been obtained, the women were interviewed using a structured questionnaire that collected information on demography, ANC utilization, pregnancy history, reported fever incidence and treatment-seeking behavior during pregnancy, knowledge and treatment of malaria, and information on household assets and characteristics.

#### In-depth interviews.

In-depth interviews were conducted with ANMs and doctors working in Murhu block. These health workers were purposively selected to include those working in subcenters a range of distances from the PHC to which they were attached, with the aim of capturing varying levels of remoteness among those interviewed.

#### Focus group discussions.

Three groups of FGDs were undertaken among pregnant women living relatively close to 1) the PHC and 2) a subcenter, and 3) with easy access to only an anganwadi center.

Participants in the IDIs gave written informed consent before starting the interview, and FGD participants gave verbal consent. One-to-one interviews were conducted for the IDIs in Hindi using a semi-structured topic guide. Focus group discussions were conducted in Hindi by a facilitator and notetaker using theme guides. In-depth interviews and FGDs aimed to assess participant’s perceptions of 1) ISTp as part of ANC and 2) components of ISTp including screening using RDTs and treatment with AS+SP (here, we report only on the first of these). In-depth interviews and FGDs were digitally recorded and transcribed verbatim.

### Data management and analysis.

Data were collected in Open Data Kit for the household surveys using tablet PCs. Data were transferred to STATA 14 (StataCorp, College Station, TX) for analysis. Analyses accounted for the survey design, adjusting for clustering within the villages used for the first stage of sample selection. Women were asked about their visits to ANC throughout the whole of their current or recent pregnancy, and therefore, data do not refer to a specific ANC visit, unless specified, but to the current or recent pregnancy as a whole.

Principal components analysis (PCA) was used to create an asset index^16^ based on household characteristics including toilet facilities; owning within the household a radio, television, mobile phone, fridge, bicycle, motorbike, car, oxcart, or auto-rickshaw; and the type of material of the floor, roof, and walls of the house. All assets were included in the PCA as binary variables.^17^ The asset index was then used to construct socioeconomic quintiles from the poorest households to the least poor.

Demographics and pregnancy history were described for pregnant women sampled in the pre- and post-implementation surveys including currently or recently pregnant, gestational age, maternal age, ever given birth, ever had a miscarriage/abortion, ever had a stillbirth, parity, the level of education, socioeconomic status, household size, religion, and primary language. Pearson’s design–based *F* test was used to test the significance of the differences in proportions between surveys for a number of outcomes including attendance at ANC, receipt of malaria tests, number of malaria tests received, any illness, fever, stated reason for having a malaria test, and having had a positive malaria test.

Univariate (unadjusted) logistic regression was used to test for an association between having a malaria test at any visit to ANC during the current or recent pregnancy and sociodemographic and pregnancy-related factors including gestational age, maternal age, level of education, religion, socioeconomic status, ever given birth, parity, ever had a miscarriage/abortion, ever had a stillbirth, had a fever during the current or recent pregnancy, attended an ANC because of illness at least once during the current or recent pregnancy, number of ANC visits, and place of the last ANC visit. Associations were tested with an adjusted Wald test. Any factors with an odds ratio (OR) significant at the 10% level (*P* < 0.1) were included in the multivariable (adjusted) logistic regression model to determine which potential predictors remained associated with having a malaria test at ANC when adjusted for other predicators. In the multivariable models, predictors were considered significant at the 10% level at all stages of model building except for the final model where *P* < 0.05 was used. The univariate and multivariable analyses were conducted for both pre- and post-implementation surveys.

The IDIs and FGDs were entered into NVivo (QSR International, Melbourne, Australia) version 11 for data management and analysis. Data were coded by one investigator (J. H.) using a combination of predefined themes developed by J. H. and J. W. Coding was reviewed by J. W., and any discrepancies were discussed and agreement reached.

### Ethics approvals.

Approval for the conduct of the study was obtained from Ethics Committees of the National Institute for Malaria Research, Delhi, and of the London School of Hygiene and Tropical Medicine (No. 6017). In addition, approval to conduct the trial was obtained from the Government of India Health Ministry Screening Committee and from the Jharkhand state Ministry of Tribal Welfare.

## RESULTS

A total of 1,087 questionnaires were completed, 553 pre- and 534 post-implementation of ISTp. In-depth interviews were conducted with 29 health workers including 24 ANMs, four medical doctors, and one member of the DHMT. A total of 13 FGDs were conducted with currently and recently pregnant women.

### What were the changes in ANC attendance, screening, and/or testing for malaria after implementation of ISTp?

The proportion of sampled women was comparable between the pre- and post-implementation surveys (23.7% versus 31.1% currently pregnant; and 76.3% versus 68.9% recently pregnant, respectively) (Table 1). The distribution of sociodemographic characteristics was similar between the two survey populations, although a higher proportion of women did not know their age at the post-implementation survey (15.7% versus 42%). Across both surveys, approximately two-thirds of women were in their third trimester. Approximately 90% of women had previously given birth, and among these, almost two-thirds, multiple times. A high proportion of women in the pre-implementation and post-implementation surveys (45.9% and 38.2%, respectively) had received no education.

**Table 1:** Characteristics of the pregnant women who participated in pre- and post-intervention household surveys

	Household survey					
	Pre-intervention (*N* = 553)			Post-intervention (*N* = 534)		
	*N*	%	95% CI	*N*	%	95% CI
Currently pregnant	131	23.7	20.7, 27.0	166	31.1	26.7, 35.8
Recently pregnant	422	76.3	72.3, 79.4	368	68.9	64.2, 73.3
Gestational age (months)						
3–6	48	36.6	25.7, 49.2	59	36.2	28.8, 44.3
7–9	83	63.4	50.8, 74.3	104	63.8	55.7, 71.2
Maternal age (years)						
15–24	214	38.7	31.7, 46.2	183	34.3	27.9, 41.3
25–34	226	40.9	35.1, 46.9	111	20.8	16.2, 26.2
35+	26	4.7	3.2, 6.9	16	3.0	1.8, 4.8
Do not know	87	15.7	10.8, 22.4	224	42.0	33.2, 51.2
Ever given birth						
Any birth	507	91.7	88.1, 94.3	475	89.0	84.9, 92.9
At least one miscarriage	46	9.1	6.4, 12.9	70	14.7	11.9, 18.1
At least 1 stillbirth	37	7.4	5.3, 10.1	17	3.6	2.1, 6.1
Parity						
Primiparous	181	35.7	29.2, 42.8	174	36.6	31.9, 41.7
Multiparous	258	50.9	45.1, 53.9	237	49.9	45.9, 53.9
Grand multiparous	68	13.4	9.2, 19.1	64	13.5	9.4, 18.9
Education						
None	254	45.9	37.0, 55.2	204	38.2	30.7, 46.4
Primary school	71	12.8	9.7, 16.8	79	14.8	11.4, 19.0
Middle school	94	17.0	13.3, 21.5	72	13.5	10.6, 17.0
High school	114	20.6	14.5, 28.5	155	29.0	21.8, 37.6
University	20	3.6	1.7, 7.4	24	4.5	2.6, 7.6
Socioeconomic status						
Least poor	111	22.1	17.2, 27.9	107	20.2	16.2, 25.0
Less poor	122	22.1	17.2, 27.8	108	20.5	15.7, 26.4
Poor	99	17.9	14.0, 22.6	107	20.0	15.8, 25.1
Very poor	116	21.0	15.8, 27.3	107	20.0	15.1, 26.1
Most poor	105	19.0	12.6, 27.5	105	19.7	13.8, 27.2
Religion						
Hindu	117	21.2	12.3, 34.0	135	25.3	16.1, 37.4
Sarna	273	49.4	37.3, 61.5	155	29.0	19.9, 40.2
Christian	152	27.5	19.0, 38.1	234	43.8	32.7, 55.6
Muslim	11	2.0	0.8, 5.3	10	1.9	0.6, 5.7
Language						
Hindi	41	7.4	4.2, 12.9	46	8.6	4.2, 16.8
Mundari	419	75.8	62.0, 85.7	373	70.0	56.5, 80.5
Sadri	85	15.4	8.6, 26.0	96	18.0	10.8, 28.4
Other	8	1.5	0.6, 3.5	19	3.6	1.7, 7.2

* Only 1.6% of women across the two surveys were not married; this variable was, therefore, not included.

The proportion of women who visited ANC at least once was not statistically different between surveys, but the number of visits to ANC tended to be higher in the post-implementation survey (Table 2). The proportion of women who visited ANC because of an illness was higher in the post-implementation survey than that in the pre-implementation survey (29.1% versus 19.7%; *P* = 0.003), and the proportion of women who had fever at least once during their pregnancy was also higher in the post-implementation survey (25.5% versus 20.6; *P* = 0.1). The proportion of pregnant women who received an RDT for malaria at ANC at least once during their current or recent pregnancy increased from pre- to post-implementation surveys (19.2% versus 42.5%; *P* < 0.0001), and the proportion of women who had more than one RDT during their current or recent pregnancy also increased (16.5%; 95% CI: 13.1, 20.5 versus 27.7%; 95% CI: 23.0, 33.0; *P* = 0.0008). The mean number of ANC visits during pregnancy for women sampled in both surveys was 3.2, and the proportion of women who had three RDTs during their current or recent pregnancy increased between pre- and post-implementation surveys (1.8%; 95% CI: 1.0, 3.1 versus 6.9%; 95% CI: 4.5, 10.3; *P* < 0.0001), respectively.

**Table 2:** Malaria screening, diagnosis, and treatment during the current or previous pregnancy pre- and post-intermittent screening and treatment during pregnancy implementation

	Household survey						
	Pre-intervention (*N* = 553)			Post-intervention (*N* = 534)		
	*N*	%	95% CI	*N*	%	95% CI	*P*-value
Visited ANC at least once	511	92.4	88.0, 95.3	489	91.6	97.3, 94.5	0.7
Number of visits to ANC							
1	32	6.5	4.3, 9.8	45	9.7	6.2, 14.9	**0.04**
2	84	17.0	13.4, 21.4	75	16.2	12.6, 20.6	
3	94	19.1	15.5, 23.2	60	13.0	9.7, 17.3	
4+	279	56.6	50.4, 62.6	282	61.0	54.0, 67.6	
At least 1 ANC visit due to illness	98	19.7	15.8, 24.3	142	29.1	25.3, 33.3	**0.003**
Fever at least once	114	20.6	16.0, 26.2	136	25.5	22.5, 28.8	0.1
At least 1 malaria RDT at ANC	98	19.2	14.9, 24.3	208	42.5	36.6, 48.7	**< 0.0001**
Number of RDT tests at ANC*							
0	398	80.7	75.5, 85.1	261	55.9	49.5, 62.1	**< 0.0001**
1	64	13.0	9.4, 17.7	125	26.8	22.2, 31.9	
2	22	4.5	2.9, 6.8	49	10.5	8.0, 13.7	
≥ 3	9	1.8	1.1, 3.1	32	6.7	4.5, 10.3	
Reason for the last malaria test*							
Illness	64	67.4	54.9, 77.8	11	19.3	12.6, 28.4	**< 0.0001**
Routine	26	27.4	17.9, 39.4	43	75.4	64.4, 83.9	
Woman requested a test	5	5.3	2.2, 11.9	3	5.3	1.4, 18.0	
Malaria test positive	37	38.5	26.9, 51.7	57	29.4	22.7, 37.1	0.1
Treated for malaria at least once	61	11.0	8.0, 15.1	65	12.2	9.7, 15.1	0.7
How many times taken treatment for malaria							
1	39	7.1	4.8, 10.2	53	9.9	8.1, 12.1	**0.04**
2	13	2.4	1.4, 4.1	12	2.2	1.1, 4.6	
3+	8	1.4	0.7, 2.9	0	0	–	
Malaria test at last ANC	23	4.5	3.1, 6.6	73	15.3	12.5, 18.5	**< 0.0001**

ANC = antenatal care; RDT = rapid diagnostic test. Bold indicates *P* ≤ 0.05.

* Those reporting “do not know” were excluded.

The proportion of pregnant women who reported that the reason for their last RDT test at an ANC clinic was because of an illness decreased from the pre- to post-implementation surveys (67.4% versus 19.3%; *P* < 0.0001), whereas the proportion reporting that the RDT test was “routine” increased (27.4% versus 75.4%; *P* < 0.0001). The proportion of women who reported that they had received an RDT test on their last visit to ANC increased from pre- to post-implementation (4.5% versus 15.3%; *P* < 0.0001). Approximately a third of pregnant women who reported to have been tested for malaria also reported that they had at least one positive test for malaria during their current or recent pregnancy. There was no change between pre- and post-implementation surveys in the proportion of pregnant women who reported that they were malaria positive or treated for malaria at least once during their current or previous pregnancy.

In univariate analyses, having at least one RDT at any visit to ANC during the current or recent pregnancy was associated with the gestational age, religion, history of fever during the pregnancy, place of ANC visit, and number of ANC visits (Table 3). In the multivariable analyses, having at least one RDT on any visit to an ANC during the current or recent pregnancy was associated with a history of fever at least once during pregnancy with an adjusted OR of 2.9 (95% CI: 1.3, 6.5).

**Table 3:** Predictors of having had at least one malaria test at ANC post-implementation of intermittent screening and treatment during pregnancy

	End line				
		Unadjusted		Adjusted	
	*N*	OR (95% CI)	*P*	Adjusted OR (95% CI)	*P*-value
Currently pregnant	50	1.0	0.1		
	158	0.8 (0.5, 1.1)			
Gestational age (months)					
3–6	8	1.0	**0.03**	1.0	0.5
7–9	42	2.6 (1.1, 6.1)		1.4 (0.5, 4.2)	
Education					
None	63	1.0	0.1		
Primary	24	1.0 (0.6, 1.6)			
Middle	27	1.2 (0.6, 2.4)			
High school	84	2.1 (1.2, 3.7)			
Universal	10	1.5 (0.6, 3.8)			
Religion					
Hindu	72	1.0	**0.0003**	1.0	0.7
Sarna	67	0.6 (0.4, 0.9)		0.7 (0.3, 2.0)	
Christian	66	0.4 (0.2, 0.6)		0.5 (0.2, 1.6)	
Muslim	3	0.3 (0.06, 1.1)		0.4 (0.03, 4.4)	
Socioeconomic group					
Least poor	51	1.0	0.3		
Less poor	43	0.7 (0.3, 1.8)			
Poor	40	0.7 (0.3, 1.6)			
Very poor	44	0.7 (0.3, 1.4)			
Poorest	30	0.4 (0.2, 0.1)			
Ever given birth					
No	21	1.0	0.8		
Yes	187	1.1 (0.6,2.0)			
Parity					
Primiparous	71	1.0	0.3		
Multiparous	98	1.0 (0.7, 1.4)			
Grand	18	0.6, (0.3, 1.2)			
Ever had miscarriage/abortion					
No	158	1.0	0.7		
Yes	29	1.1 (0.6, 2.0)			
Ever had stillbirth					
No	178	1.0	0.4		
Yes	9	1.7 (0.5, 5.6)			
Had a fever at least once during pregnancy					
No	117	1.0	**< 0.0001**	1.0	**0.01**
Yes	91	3.9 (2.5, 6.0)		2.9 (1.3, 6.5)	
Place of ANC visit					
Hospital	85	1.0	**0.003**	1.0	0.09
Primary health center/CHC	31	1.0 (0.4, 2.3)		0.3 (0.06, 1.5)	
Subcenter	9	0.9 (0.3, 2.5)		4.7 (0.9, 23.2)	
Anganwadi	83	0.4 (0.3, 0.7)		0.7 (0.3, 1.7)	
Number of ANC visits					
0	–	–			
1 or 2	30	1.0	**< 0.0001**	1.0	0.09
2					
3	14	0.8 (0.4, 1.8)		1.5 (0.4, 6.0)	
4 or more	152	3.7 (2.2, 6.1)		4.3 (1.2, 15.5)	

ANC = antenatal care; CHC = community health center; OR = odds ratio. Bold indicates *P* ≤ 0.05.

### What were the perspectives of health workers and pregnant women on screening for malaria with RDTs at each visit to ANC?

Health workers reported on both their own perspectives of screening pregnant women for malaria at ANC and their perceptions of pregnant women’s feelings on this topic (Table 4).

**Table 4:** Acceptability of ISTp by health workers and pregnant women

Theme	Subthemes
	To what extent do health workers accept ISTp?
Screening for malaria during ANC	Positive
	Testing all pregnant women is best as pregnant women do not always show symptoms when they have malaria
	ISTp should continue, good to identify malaria and give treatment
	ISTp is best because you can get malaria anytime in pregnancy
	Easy for health worker and pregnant woman to know malaria status
	Negative
	Only pregnant women with complaints/symptoms should be tested
	Pregnant women don’t need to be tested in the last month of pregnancy
RDTs	Positive
	Results are fast
	We trust the RDT results
	Negative
	Results are not always reliable/accurate
	Pregnant women who test negative but have symptoms are referred to hospital for further checks
	Sometimes results need to be confirmed with blood slides
	To what extent do health workers perceive that pregnant women accept ISTp?
Screening for malaria during ANC	Positive
	Pregnant women are happy to be tested
	Initially they did not like it, but now, they have awareness about it, and they want the test
	Negative
	They do not like giving blood samples
	Fear blood testing will reduce blood
	They do not feel they need testing if they feel fine, and we have to convince them
	Because of lack of understanding—some do not like it, they do not understand its importance
	Pregnant women are not especially interested in malaria testing
RDTs	Positive
	Pregnant women prefer RDTs
	Pregnant women prefer microscopy (1 provider)
	To what extent do pregnant women accept ISTp?
Screening for malaria during ANC	Positive
	Like to know if I have malaria
	Service is better; now we get more tests
	Negative
	It was painful
	It made me afraid, uneasy
	I felt weak, dizzy

ANC = antenatal care; ISTp = intermittent screening and treatment during pregnancy; RDT = rapid diagnostic test.

### Perceptions of health workers and pregnant women on screening pregnant women for malaria at ANC.

Overall health workers were positive about screening pregnant women for malaria at ANC; however, they perceived pregnant women to be negative about this screening. There was a strong belief that the major symptom of malaria is fever, and there was no suggestion that any other symptom was important. However, several health workers suggested that screening pregnant women for malaria is important because many women who have malaria are not symptomatic. For most of them, this meant presenting with fever; however, there was also a sense that malaria was changing and that fever was not as prevalent in MiP as it was previously.

“For all pregnant women. As malaria is not visible. And for a few, they do not know if they have malaria. These days malaria is changing its form too. Some have fever, some do not have fever. If she has fever and we do not know, then we may check by kit [RDT].” IDI 20_ANM

“This is a malaria prone area. Here there are a lot of cases where the patient is not able to tell. This is an anemic area too. Testing here is really important. IST should be done......it should be continued.” IDI 26_Doctor

Health workers were aware of the need to protect pregnant women from malaria to ensure the health of both the mother and baby. The health workers believed in the link between malaria and anemia, which was perceived as an important health problem in the study area.

“We want that the test should be done. As we prevent her…from malaria she might develop anemia and it may be a cause of death of both child and mother. With the test we may prevent these deaths.” IDI 24_ANM

There were a few ANMs who felt that pregnant women should only be tested for malaria when symptomatic. This was because they felt that pregnant women openly shared information about their symptoms and that having the tests could result in women not wanting to attend ANC.

“I think would be better to do IST for women having symptoms. Women definitely share their problem, like feeling of fever or any other symptoms, if tests would be done for them, then easily [we] would get the result.” IDI 1_ANM

“No. Those who do not have fever, it [malaria test on every ANC visit] is not good to be done in them.” IDI 15_ANM

Although there were some positive perceptions by health workers on how pregnant women reacted to being screened for malaria at ANC, there were also many that were negative. Health workers perceived pregnant women to feel that they were getting a good service at ANC if they were given many tests and that this encouraged them to attend ANC. Other health workers reported that pregnant women did not like having blood tests as they “feared a reduction in their blood.”

“We normally do the test. We have got yellow cards accordingly we are doing, but we are not doing repeatedly, [we are] doing for 4 times only. [A] few women say that you are taking blood every time and it’s reducing [blood].” IDI 8_ANM

“Yes, they like it [malaria testing] but they say do not do it again and again. Every day you do the checkup, it hurts the hand...” IDI 24_ANM

“It [number of women] has increased a lot. Earlier we did not use to come as they used to think that they have to go just for injection. Now since the malaria testing has begun…in every month they come for testing.” IDI 22_ANM

Responses from pregnant women on testing at ANC were positive with both “knowing the level of blood” and “knowing if you have malaria” being mentioned as important. Many women reported that having the blood test was slightly painful.

“Participant (P)6: I was tested in xxx, it was slightly painful, like an ant bite.

P5: In the hospital blood test was there, they had taken blood from the finger. It was slightly painful.

P7: I was tested in … hospital … it was painful even for me.

P3: For me it was in xxx, it was slightly painful in the finger.” FGD 10

Despite the pain, there was a sense from the pregnant women involved in the FGDs that the introduction of blood tests in ANC was good because it was accompanied by increased sharing of information. However, most women said that they were either given only one blood test or not given a blood test at all.

“P: There are differences; initially blood samples were not collected. We were not explained about diseases, but now a days all check-ups are done and we get all information.” FGD 1

### Perceptions of health workers and pregnant women on RDTs.

According to health workers interviewed, the main benefit to using RDTs was the speed at which they received the parasitological diagnosis of malaria that meant, when positive, treatment could be initiated quickly. This view was widespread among ANMs.

“Kits are good because [they] quickly give the report. Through slidse it takes time, within this period her malaria will increase.” IDI 4_ANM

Despite the positive views regarding the speed of diagnosis with RDTs, there was widespread doubt about the accuracy of the result, which for some led to additional testing with microscopy for confirmation. At the lower level health facilities, there was almost universal acknowledgment that if the RDT result was in doubt and the woman appeared symptomatic, a referral to a higher level health facility was made.

## DISCUSSION

This study was an evaluation of a 1-year trial implementation of ISTp in one subdistrict in Jharkhand state. The proportion of pregnant women tested for malaria at least once during a routine ANC visit increased post-implementation of the ISTp strategy. However, this amounted to less than half of pregnant women sampled. Despite a mean of 3.2 visits to ANC during pregnancy, post-implementation of the ISTp intervention, only 8% of women who had completed their pregnancy received an RDT on each of three visits to ANC. The IDIs and FGDs offer an explanation for this finding. Although health workers were generally positive in their perceptions of screening pregnant women for malaria at ANC, their perceived understanding of the views of pregnant women on this intervention was relatively negative. Health workers believed that pregnant women did not like having blood withdrawn and would, therefore, not want to be tested for malaria on every visit to ANC. Pregnant women themselves, as noted in the FGDs, were less negative about receiving blood tests, but there was a feeling that being tested for malaria on every ANC visit was too much. Focus group discussions require that participants are comfortable in their surroundings and with the other members of the group^18^; it is possible that pregnant women were more likely to express their concerns to individual health workers in ANC than to strangers in an FGD. Most, but not all, pregnant women reported not having been tested or having been tested only once. Frontline health workers must make decisions based on their knowledge of the health benefits of the interventions they offer but in the context of the reactions of their clients. For example, although knowing the health benefits of intermittent preventive treatment in pregnancy, health workers did not offer this intervention to pregnant women who had not eaten before attending ANC because of the mild side effects such as vomiting and nausea when taking SP on an empty stomach.^19^

The proportion of women who reported that they were malaria positive or treated for malaria was the same in the pre- and post-implementation surveys. This is not unexpected as the prevalence of malaria was found to be very low in this setting (< 5.0% based on testing 3,300 women in a neighboring district^12^), and less than half of women were tested, and this indicator was based on reports of women.

Health workers expressed support for ISTp in this study primarily because they believed that not all pregnant women with malaria were symptomatic, which in this context meant febrile. These beliefs are supported by the findings of the randomized trial on ISTp versus PCD,^12^ and similar findings have recently been reported in Indonesia.^9^ Passive case detection as a strategy requires that malaria is symptomatic and, therefore, is not effective in contexts such as Eastern India, where a large number of cases of malaria are, in fact, asymptomatic. It is important to detect asymptomatic infections as they may reflect placental infection. There is reasonable evidence that a positive RDT or blood film is a predictor of placental malaria. Passive case detection also requires that if a woman is symptomatic, these symptoms are either reported to, or picked up by, health workers. There was disagreement among health workers in this study as to whether pregnant women voluntarily reported their symptoms to health workers. There are, however, previous reports from Eastern India that pregnant women do not necessarily freely inform health workers of their symptoms.^20^

Being tested for malaria at least once during pregnancy in this study population was associated with having a history of fever at least once during pregnancy. This suggests that although there was a belief among health workers that malaria was asymptomatic in many pregnant women, this concept had not translated to implementation of ISTp, and testing was driven more by symptoms than by ISTp. The finding of a recent trial of ISTp in neighboring districts of Jharkhand state^12^ that ISTp detected more malaria infections than PCD is dependent on the fidelity with which the ISTp strategy is implemented. The results of this study suggest that a reduced number of infections will be detected during routine program implementation than was found in the trial setting unless attention is paid to improving the adoption of the strategy by health workers.

There were several limitations to this study. Respondents replying “don’t know” to many of the questions was not uncommon, which resulted in missing data. The denominators across the variables are, therefore, not constant. For example, over 40% of women interviewed in the post-implementation survey did not know their age. However, this does suggest that where women did not know the answer, they were not worried about saying so, and this may indicate that when they did respond, they were confident in their response, which can, therefore, be relied on. Asking about the whole period of the current or recent pregnancy rather than one particular ANC visit will have reduced the specificity of some of the responses. The study design was such that changes in the proportion of pregnant women tested for malaria pre- and post-implementation of ISTp was measured, but the observed changes cannot be directly attributed to the implementation of the intervention. However, we are not aware of any other interventions during the same time period that could have contributed to the changes measured.

## CONCLUSION

Within the routine health system, the proportion of pregnant women tested for malaria at least once during their pregnancy increased post-implementation of ISTp, but this was the case for less than half of ANC visits. Just 8% of pregnant women were screened for malaria on each of the three visits to ANC. Intermittent screening and treatment during pregnancy was not sufficiently adopted by health workers to ensure the increased detection of malaria infections achievable with this strategy in this setting.
